# RVLM C1 Neurons Innervate Sacral as well as Thoracolumbar Autonomic Preganglionic Neurons in the Rat

**DOI:** 10.1002/cne.70134

**Published:** 2026-01-31

**Authors:** I. J. Llewellyn‐Smith, L. Travis, A. A. Connelly, J. K. Bassi, C. Menuet, A. M. Allen

**Affiliations:** ^1^ Cardiovascular Medicine, Human Physiology and Centre for Neuroscience, College of Medicine and Public Health Flinders University Bedford Park South Australia Australia; ^2^ Department of Anatomy and Physiology University of Melbourne Victoria Australia; ^3^ INMED, INSERM, Aix Marseille University Marseille France; ^4^ Florey Institute of Neuroscience and Mental Health University of Melbourne Victoria Australia

**Keywords:** adrenergic, cocaine‐ and amphetamine‐regulated transcript (CART), intermediolateral cell column, micturition, Onuf's nucleus, parasympathetic preganglionic neuron, phenylethanolamine N‐methyltransferase (PNMT), rostral ventrolateral medulla, somatic motor neurons, spinal cord

## Abstract

We examined the distribution of axons throughout the spinal cord of the rat that were either immunoreactive for the adrenaline‐synthesizing enzyme, phenylethanolamine N‐methyltransferase (PNMT), or derived from medullary C1 neurons, one of the three groups of neurons in the brain that synthesize PNMT. We observed that PMNT‐immunoreactive axons, as well as C1 axons labelled with GFP from viral transduction, innervate most, but not all, sympathetic preganglionic neurons in the thoracolumbar spinal cord. GFP‐positive C1 axons provided innervation to sympathetic preganglionic neurons that expressed cocaine and amphetamine regulated transcript, an accepted marker of sympathetic vasomotor neurons. In addition, we observed axons from PNMT‐containing and C1 neurons caudal to the distribution of sympathetic preganglionic neurons in the sacral spinal cord where they closely apposed parasympathetic preganglionic neurons retrogradely labelled from the major pelvic ganglion. We also found close appositions from PNMT‐immunoreactive or GFP‐labelled C1 axons on choline acetyltransferase‐stained parasympathetic preganglionic neurons activated by the micturition reflex, thus providing clear evidence of a non‐cardiovascular target for RVLM C1 neurons. Furthermore, we observed a few PNMT‐positive and GFP‐positive C1 axons making close appositions with somatic motor neurons in Onuf's nucleus in the sacral cord and in the ventral horn at more rostral levels. These data provide a comprehensive map of the distribution of adrenergic inputs to the spinal cord and identify parasympathetic preganglionic neurons, including those involved in the micturition reflex, as well as sympathetic preganglionic neurons as the major targets for these inputs.

## Graphical abstract

Axons expressing the adrenaline‐synthesizing enzyme, phenylethanolamine N‐methyltransferase (PNMT), project widely throughout the spinal cord. Many of these axons are from C1 neurons of the rostral ventrolateral medulla. We confirm that dense arrays of PNMT‐expressing, C1‐derived axons surround thoracolumbar sympathetic preganglionic neurons, many of which are putative vasomotor neurons. The C1/PNMT axons are also more widespread, innervating sacral preganglionic neurons activated by the micturition reflex and, very sparsely, somatic motor neurons in the ventral horn.

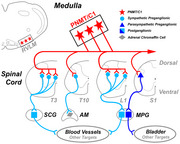

## 1 Introduction

The distribution of CNS neurons that express the enzymes required for catecholamine synthesis was first described in the 1970s. Three catecholamine‐synthesizing cell groups located in the medulla oblongata proved to be unique because they express all of the catecholamine‐synthesizing enzymes required to produce adrenaline (Hokfelt et al. 1973). Thus, these neurons can be unequivocally defined by their expression of phenylethanolamine N‐methyltransferase (PNMT), the enzyme responsible for the conversion of noradrenaline to adrenaline (Axelrod 1962). These medullary cell groups were named C1, C2, and C3.

Most of the interest in medullary adrenergic neurons has focused on C1 neurons, which form a column in the ventral medulla and can be loosely divided into two groups based upon their axonal projections. Neurons in the rostral part of the C1 column project to the spinal cord, whereas C1 neurons in the caudal part of the column send axons to other locations within the medulla and to more rostral CNS sites (Verberne et al. 1999). The bulbospinal C1 neurons provide monosynaptic innervation to sympathetic preganglionic neurons (SPN) in the spinal cord, and can be trans‐synaptically labelled using viral tracers injected into a variety of sympathetically innervated viscera (Sly et al. 1999; Strack et al. 1989). While C1 neurons express the enzymes required for catecholamine production and the vesicular monoamine transporter type 2 (Sevigny et al. 2008), they also express vesicular glutamate transporter type 2 (VGLUT2) and are considered excitatory glutamatergic neurons (Stornetta et al. 2002). The spinally projecting C1 neurons occur in the rostral ventrolateral medulla (RVLM), a region where neuronal inhibition induces dramatic decreases in sympathetic vasomotor activity and blood pressure (Dampney 1994). More recent studies indicate that the rostral C1 neurons do not play a major role in determining resting sympathetic tone and blood pressure (Madden and Sved 2003; Marina et al. 2011; Schreihofer and Guyenet 2000; Wenker et al. 2017) but transmit respiratory‐modulated sympathoexcitatory activity that is involved in the development of high blood pressure (Menuet et al. 2017). The bulbospinal C1 neurons are activated by multiple stimuli and in turn modulate sympathetic efferent activity involved in blood pressure regulation (Guyenet et al. 2013).

There is now considerable evidence showing that C1 neurons are not exclusively involved in cardiovascular regulation. Many of the targets of axonal projections from C1 neurons do not have an obvious cardiovascular function and are not directly connected to sympathetic efferent pathways. Some C1 neurons project to different higher brain centers, including neuroendocrine nuclei of the hypothalamus (Card et al. 2006; Sevigny et al. 2008). Selective stimulation of C1 neurons activates parasympathetic preganglionic neurons (PPN) in the dorsal motor nucleus of the vagus (DePuy et al. 2013) and noradrenergic neurons in the locus coeruleus (Holloway et al. 2013), increases arousal (Souza et al. 2020), and results in sleep‐state‐dependent effects on respiratory function (Burke et al. 2014; Toledo et al. 2022). Activation of C1 neurons is a necessary component of the neural pathway by which restraint stress can protect the kidney against ischemia‐reperfusion injury (Abe et al. 2017). Furthermore, even spinally projecting C1 neurons are involved in the regulation of non‐cardiovascular functions such as glucose homeostasis (Ritter et al. 1998; Verberne and Sartor 2010). Together, these data point to a more widespread role for C1 neurons in both physiology and pathology.

In comparison to the C1 neurons, the C2 and C3 neuron groups are poorly understood, and are mostly studied incidentally because of their localization in the same rostrocaudal planes as the C1 neurons. The C2 group occurs in the rostral nucleus of the solitary tract and does not have a strong axonal projection to the spinal cord, so it is not of interest for this manuscript. The C3 group is diffusely distributed in the rostral dorsomedial medulla and can be divided into four distinct columns of neurons. Like the C1 groups, some of these neurons project rostrally, while the neurons in the medial and median columns project to the spinal cord (Menuet et al. 2014; Sevigny et al. 2017). Largely due to their diffuse distribution, ascribing a function to C3 neurons has proved difficult. Nevertheless, optogenetic approaches have shown that the spinally projecting C3 neurons are sympathoexcitatory and activated by glucoprivation, but not hypotension (Menuet et al. 2014; Ritter et al. 1998). Like C1 neurons, C3 neurons also express VGLUT2 and are considered excitatory glutamatergic neurons (Stornetta et al. 2002).

Similar to the widespread distribution of C1 projections throughout the CNS (Card et al. 2006), we have previously reported that C1 neurons project to other spinal regions in addition to areas containing SPN (Sevigny et al. 2008). In the current study, we have examined the spinal distribution of PNMT‐immunoreactive axons and spinal C1 axons identified by anterograde tracing from injections of PRSx8‐GFP into the RVLM. These two methods have allowed us to map the spinal projections of C1 neurons in detail for the first time. In studies combining labelling of C1 axons with functional neuroanatomy for spinal neurons controlling bladder function, we provide clear anatomical evidence for the involvement of C1 neurons in functions bearing little relationship to blood pressure control.

## 2 Methods and Materials

All experiments were conducted in accordance with the Australian National Health and Medical Research Council “Guidelines to Protect the Wellbeing of Animals used for Scientific Purposes” and were approved by the Animal Welfare Committee of Flinders University, and by the Animal Experimentation Committee of the University of Melbourne. The use of recombinant viruses was approved by the University of Melbourne Biosafety Committee.

### 2.1 Virus Production

To transduce RVLM C1 neurons in adult male Sprague Dawley rats (250–350 g), recombinant lentivirus was injected using published protocols (Chen et al. 2010; Sevigny et al. 2008). Rats were anesthetized with a mixture of ketamine (60 mg/kg i.m., Lyppard, Victoria, Australia) and medetomidine (250 µg/kg i.m., Pfizer Animal Health, NSW, Australia), and lentivirus expressing green fluorescent protein (GFP) under the control of the PRSx8 promoter (Hwang et al. 2001) was injected into the RVLM, using published protocols (Menuet et al. 2014). The selectivity of this promoter/virus combination for transducing C1 neurons in the RVLM has been extensively documented, including demonstration of vesicular monoamine transporter 2 in the axonal terminals of transduced C1 neurons (Card et al. 2006; Sevigny et al. 2008). Although some retrotrapezoid nucleus neurons are transduced within the region of the injection site, the axonal projections of these neurons are confined to the lower brainstem (Abbott et al. 2009).

### 2.2 Retrograde Tracing

In all experiments, we used cholera toxin B subunit (CTB; 0.5% in filtered deionized water; List Biological Laboratories, Campbell, CA, USA) for retrograde tracing. To identify neurons projecting to different prevertebral ganglia, male Sprague Dawley rats were anesthetized with 3% isoflurane (Rhodia Australia, Victoria, Australia) in oxygen before injecting CTB into the right superior cervical ganglion (SCG; *n* = 6 rats; 5 µL per animal), the left adrenal medulla (AM; *n* = 4 rats; 10 µL per animal), the coeliac ganglion (CG; *n* = 3 rats; 10 µL per animal), or the left major pelvic ganglion (MPG; *n* = 4 rats; 5 µL per animal). Rats recovered in their home cage for 3–7 days after injection, to allow for retrograde transport of the CTB, and were then perfused.

### 2.3 Micturition Reflexes

To examine neurons activated during micturition reflexes, rats were anesthetized with urethane (1.5 g/kg s.c.; Sigma‐Aldrich, NSW, Australia) and a local anesthetic (bupivicaine 5 mg/mL; Pfizer Animal Health) applied topically to the skin of the lower abdomen. The bladder was exposed through an incision in the skin and muscle of the lower abdomen; then, 2 × 25‐gauge needles were inserted into the dome of the bladder. Saline was infused through one needle with an infusion pump (120 µL/min), and the other needle was connected to a pressure transducer. Once the needles were in place, the saline infusion continued for 90 min. Control rats were treated identically, but without the bladder infusion. Thirty minutes after cessation of the infusion (i.e., 2 h after the beginning of the infusion), rats were perfused while still under urethane anesthesia.

### 2.4 Tissue Preparation

Those rats not already anesthetized with urethane were deeply anesthetized with pentobarbitone (100 mg/kg, i.p.). Both urethane‐anesthetized and pentobarbitone‐anesthetized rats were perfused for immunohistochemistry. The abdominal cavity was opened, and the renal arteries and intestines were clamped with hemostats. The heart was exposed, and a needle was inserted through the left ventricle into the ascending aorta. Following administration of 1 mL of heparin (1000 IU/mL; Hospira, Melbourne, Australia), the rats were perfused with 500 mL of DMEM/F12 culture medium (Dulbecco's modified Eagle's medium, Catalogue # D8900, Sigma‐Aldrich, St. Louis, MO, USA) and then 1 L of 4% formaldehyde in 0.1 M phosphate buffer, pH 7.4. The spinal cords and brainstems were removed and postfixed for 3–5 days on a shaker at room temperature in the same fixative as for perfusion. We prepared spinal cord blocks that contained three to four spinal segments; we used a brain matrix (Braintree Scientific, Braintree, MA, USA) to prepare brainstem blocks. All blocks were infiltrated with 20% and then 30% sucrose. The spinal cord blocks were sectioned horizontally at 25 µm on a cryostat into two series of alternate sections; brainstem blocks were cut coronally into four series of 30‐µm sections.

### 2.5 Immunohistochemistry

All sections for immunohistochemistry were washed, 3 × 10 min, in 10 mM Tris, 0.9% NaCl, and 0.05% thimerosal in 10 mM phosphate buffer, pH 7.4, (TPBS) containing 0.3% Triton X‐100 and then exposed to 10% normal horse serum (NHS; Gibco Heat‐Inactivated Horse Serum; Life Technologies, Mulgrave, Australia) in TPBS‐Triton for 30 min. We used 10% NHS‐TPBS‐Triton to dilute primary antibodies, 1% NHS‐TPBS‐Triton to dilute secondary antibodies, and TPBS‐Triton to dilute the ExtrAvidin‐peroxidase complex. We purchased all of the biotinylated secondary antibodies used in this study from Jackson ImmunoResearch Laboratories (West Grove, PA, USA) and used them all at a dilution of 1:500. All sections were exposed to a 1:1500 dilution of ExtrAvidin‐horseradish peroxidase (ExtrAvidin‐HRP; RRID:AB_2620165; Catalogue # E‐2886, Sigma‐Aldrich, St. Louis, MO, USA) after incubation in biotinylated secondary antibodies. All incubations in immunoreagents occurred at room temperature with constant agitation on a shaker. After each incubation in an immunoreagent, sections were washed, 3 × 10 min, in TPBS. The immunostained sections were mounted in serial order on slides coated with chrome alum‐gelatine. After drying, the slides were coverslipped with Permaslip (Catalogue # A325A, Alban Scientific Inc, St. Louis, MO, USA).

*Double immunoperoxidase labelling for PNMT plus CTB:* Double immunoperoxidase labelling was used to localize PNMT in adrenergic axons and CTB in retrogradely labelled SPN on one series of each 1:2 series of spinal cord sections. After exposing sections to 10% NHS‐TPBS‐Triton, they were incubated for 2–3 days in rabbit anti‐PNMT antibody (1:5000 dilution; kind gift from Dr. Luc Denoroy; no RRID available; Table 1). After the primary antibody, the sections were incubated overnight in biotinylated donkey anti‐rabbit immunoglobulin (RRID:AB_2340593; Catalogue #711‐065‐152, Jackson ImmunoResearch Laboratories) followed by 4–6 h incubation in ExtrAvidin‐HRP. A black peroxidase reaction product was produced in PNMT‐immunoreactive axons using a cobalt‐ and nickel‐intensified diaminobenzidine (DAB) reaction (Llewellyn‐Smith et al. 2005). After PNMT visualization, the sections were blocked again in 10% NHS‐TPBS‐Triton and then incubated in goat anti‐CTB antibody (1:800,000 or 1:1,500,000 dilution; no RRID available; Catalogue #703; List Labs, Campbell, CA, USA; Table 1). After washing, the sections were incubated as above in biotinylated donkey anti‐goat immunoglobulin (RRID:AB_2340397; Catalogue #705‐065‐147, Jackson ImmunoResearch Laboratories) followed by ExtrAvidin‐HRP. An imidazole‐intensified DAB reaction was used to achieve a brown reaction product in neurons that contained retrogradely transported CTB (Llewellyn‐Smith et al. 2005).

**TABLE 1:** Primary antibodies used.

Antigen	Immunogen	Manufacturer, species antibody was raised in, mono‐ vs. polyclonal, catalog and lot number	Dilution used
Cholera toxin b subunit	b subunit isolated from *Vibrio cholerae* type Inaba 569B	List Labs (Campbell, CA, USA), Goat polyclonal, Catalogue # 703; no RRID available.	1:800,000, 1:1,000,000, or 1:1,500,000
Choline acetyltransferase (ChAT)	Human placental choline acetyltransferase	Chemicon (Temecula, CA, USA), Goat polyclonal, RRID:AB_2079751; Catalogue # AB144P	1:1,000
Cocaine‐ and amphetamine‐regulated transcript (CART)	Conserved amino acid sequence 55–102 of CART	Phoenix Pharmaceuticals Inc. (Belmont, CA, USA), Rabbit polyclonal, RRID: AB_2313614; Catalogue # H‐003‐62	1:50,000
Fos	A proprietary immunogen sequence mapping within amino acids 125–175 of c‐Fos of human origin (accession # P01100). Possibly amino acids 128–152 of this protein (Pierce et al. 1996; Wargnier et al. 1995; Welter et al. 1995), which are KVEQLSPEEEEKRRIRRERNKMAAA^1^	Santa Cruz Biotechnologies (Dallas, Texas, USA), Rabbit polyclonal, RRID:AB_2231996; Catalogue # sc‐253 (also known as K25)	1:5000
Green fluorescent protein (GFP)	Recombinant full‐length GFP corresponding to *Aequorea victoria* GFP	Abcam Inc. (Cambridge, UK), Chicken polyclonal, RRID: AB_300798; Catalogue #: ab13970	1:20,000
Phenylethanolamine N‐methyltransferase (PNMT)	Bovine adrenal PNMT	Kind gift from Dr. Luc Denoroy, Rabbit polyclonal; No RRID or Catalogue # available	1:5000

^1^ See https://www.uniprot.org/uniprot/P01100#sequences for “canonical” sequence of human c‐Fos, accession # P01100.

*Double immunoperoxidase labelling for GFP plus ChAT, CART, or CTB:* We used the same protocol as above to localize GFP in the axons of C1 neurons that had been transduced with the PRSx8‐GFP virus, plus either choline acetyltransferase (ChAT, which occurs in all SPN), cocaine‐ and amphetamine‐regulated transcript (CART, which occurs in some SPN), or CTB retrogradely transported from either the SCG, AM, CG, or MPG. After blocking with NHS, spinal cord sections from rats with PRS8‐GFP injections into C1 were exposed to chicken anti‐GFP polyclonal antibody (1:20,000; RRID: AB_300798; Catalogue # ab13970; Abcam Inc., Cambridge, UK; Table 1) in 10% NHS‐TPBS‐Triton for 3–4 days, then to donkey chicken anti‐IgY (RRID: AB_2313596; Catalogue # 703‐065‐155; Jackson ImmunoResearch Laboratories) overnight, and finally to ExtrAvidin‐HRP for 4 h. GFP‐immunoreactive axons were stained black with a nickel‐intensified DAB reaction. After staining for GFP, washing, and another NHS blocking step, markers in the cell bodies and dendrites of SPN and PPN were detected. Sections with GFP staining were exposed to a second primary antibody for 3–4 days and then to a second, biotinylated secondary antibody overnight. The primary/secondary antibody combinations used were either goat anti‐ChAT (1:1,000; RRID: AB_2079751; Catalogue # AB144P; Chemicon, Temecula, CA, USA; Table 1) and then biotinylated donkey anti‐goat IgG (RRID:AB_2340397; Catalogue # 705‐065‐147Jackson ImmunoResearch Laboratories); rabbit anti‐CART (1:50,000; RRID: AB_2313614; Catalogue # H‐003‐62; Phoenix Pharmaceuticals Inc., Belmont, CA, USA, Table 1) and then biotinylated donkey anti‐rabbit IgG (RRID:AB_2340593; Catalogue #711‐065‐152; Jackson ImmunoResearch Laboratories); or goat anti‐CTB (1:1,000,000; no RRID available; Catalogue # 703; List Labs; Table 1) and then biotinylated donkey anti‐goat IgG (RRID:AB_2340397; Catalogue # 705‐065‐147; Jackson ImmunoResearch Laboratories). Regardless of the primary/secondary antibody combination, sections were finally incubated in ExtrAvidin‐HRP for 4 h, and then markers in SPN or PPN were stained brown with an imidazole‐intensified DAB reaction.

*Triple immunoperoxidase labelling for Fos plus ChAT plus PNMT or GFP:* A triple labelling protocol based on the double labelling protocol above was used to localize Fos plus ChAT plus either PNMT or GFP in spinal cords from rats in which saline infusions into the bladder evoked micturition reflexes. Triple labelling was achieved by three successive rounds of staining to localize each antigen individually. In all cases, Fos was localized first using rabbit anti‐Fos (1:5,000, RRID:AB_2231996, Catalogue # sc‐253, Santa Cruz Biotechnologies, Dallas, Texas, USA; Table 1), biotinylated donkey anti‐rabbit IgG (RRID:AB_2340593; Catalogue #711‐065‐152; Jackson ImmunoResearch Laboratories), ExtrAvidin‐HRP, and a nickel‐DAB reaction to stain Fos‐containing nuclei black. After detection of Fos, either PNMT‐immunoreactivity or GFP‐immunoreactivity was localized using the same primary and secondary antibodies and dilutions described above, followed by ExtrAvidin‐HRP and a nickel‐DAB reaction to turn immunoreactive axons black. Finally, a third round of immunostaining detected immunoreactivity for ChAT, using the same immunoreagents and dilutions as for double staining, and then an imidazole‐DAB reaction to turn ChAT‐positive cell bodies brown.

### 2.6 Antibody Characterization

We used titrations for each different combination of primary and secondary antibodies in tissue fixed and processed as described above to determine which dilutions yielded the best specific staining while producing minimal nonspecific staining.

*Rabbit anti‐PNMT antiserum* (gifted as lyophilized aliquots by Dr. Luc Denoroy) was one of the first antibodies used to identify adrenergic neurons in the brains of laboratory mammals and humans (Carlton et al. 1989; Garcia et al. 1996; Kitahama et al. 1986; Kitahama et al. 1985; Mittendorf et al. 1988; Siaud et al. 1989). In this study, we found that the Denoroy anti‐PNMT revealed the expected distribution of PNMT‐immunoreactive axons in the rat spinal cord, and we have previously shown that it shows the expected distribution of PNMT‐immunoreactive cell bodies in the rat medulla (Menuet et al. 2014; Senthilkumaran et al. 2018). Pre‐absorption of this antibody with purified bovine PNMT has previously been shown to eliminate staining in the medulla oblongata (Carlton et al. 1987).

*Rabbit anti‐Fos antiserum* (Santa Cruz Biotechnologies [SCBT]) was raised against highly conserved amino acids 128–152 of the human c‐fos protein (Pierce et al. 1996; Wargnier et al. 1995; Welter et al. 1995). This amino acid sequence occurs within the region of human Fos protein (amino acids 125–175) that contains the immunogen for sc‐253. We have already demonstrated that overnight absorption of anti‐Fos sc‐253 with the peptide against which it was raised eradicates Fos immunostaining (Fenwick et al. 2006). SCBT's Western blotting on lysates of human transfected cells shows bands at molecular weights corresponding to the protein products of c‐fos and FosB and FRA2. We have also found that sc‐253 reveals a distribution of 2‐deoxyglucose‐activated C1 and C3 neurons in rats (Korim et al. 2016; Menuet et al. 2014) that is very similar to the distribution revealed by our Arnel anti‐Fos antiserum (Ritter et al. 1998) and to the distribution detected by others with a different SCBT anti‐Fos that was raised against amino acids 3–16 of the protein encoded by human c‐fos (Parker et al. 2015; Parker et al. 2017). The SCBT anti‐Fos sc‐253 also shows virtually the same distribution of hypotension‐responsive C1 neurons in normotensive WKY rats as our sheep anti‐Fos (Minson et al. 1996) and produces a similar staining pattern to other anti‐Fos antisera that have been used in studies on barosensitive C1 neurons in rats (Chan and Sawchenko 1998; Stornetta et al. 2002; Sved et al. 1994).

*Goat anti‐ChAT antiserum* (Chemicon‐Merck) was raised against human placental enzyme. The manufacturer verified the specificity by Western blot analysis using mouse brain lysates and detected a 68–70 kDa band corresponding to the ChAT protein. Overnight absorption with 2 µg/mL of ChAT abolishes staining in cell bodies and axons (Llewellyn‐Smith et al. 2007), and we observed that the antibody yielded a labelling pattern within rat brain sections previously reported in literature (Menuet et al. 2020; Ngo et al. 2020; Sevigny et al. 2012).

*Chicken anti‐GFP antiserum* (AbCam plc, Cambridge, UK) is raised against recombinant GFP. The manufacturer determined the specificity by Western blot analysis and immunohistochemistry using transgenic mice expressing the GFP gene product. The antibody shows labelling only in tissues expressing GFP (Bochorishvili et al. 2014).

*Rabbit anti‐CART antiserum* (Phoenix Pharmaceuticals Inc.) is a polyclonal antibody raised against a conserved sequence, amino acids 55–102, of CART. We have previously published with this antibody (Burman et al. 2004; Fenwick et al. 2006).

*Goat anti‐CTB antiserum* (List Labs) is a monoclonal antibody from the CTB isolated from *Vibrio cholerae* type Inaba 569B. It has been used extensively by us for anatomical studies for retrograde tracing (Fenwick et al. 2006; Llewellyn‐Smith et al. 2007).

### 2.7 Preparation of Figures

Sections were imaged as described previously (Senthilkumaran et al. 2018). Digital images from sections were captured using a SPOT RT color camera and SPOT RT software v3.0 (Diagnostic Instruments Inc., Sterling Heights, MI, USA). For adjustment of sharpness, brightness, and contrast, and to prepare montages and plates, .tif files were imported into Adobe Photoshop.

### 2.8 Terminology

Neurons in the lateral horn of the lumbosacral segments of the spinal cord were classified as part of the parasympathetic nervous system by Langley, based on physiological considerations and anatomical observations, such as that their axons do not enter the paravertebral chain (see Horn [2018] for a discussion). Recent studies in prenatal mice have shown that the expression of a number of genetic markers, many with undefined functions, is the same in spinal SPN and PPN (Brunet 2025; Espinosa‐Medina et al. 2016). On the basis of these results, it has been argued that, in fact, there is no sacral parasympathetic outflow. This intriguing idea has been the subject of considerable debate in the literature (Brunet 2025; Horn 2018; Janig et al. 2017; Neuhuber et al. 2017) and remains contentious. Our study does not contribute to this debate but rather examines the inputs to lumbosacral preganglionic neurons activated by the micturition reflex. Given that these neurons have long been considered PPN and are the subject of hundreds of published studies using that term (de Groat et al. 2015; Fowler et al. 2008), we have continued to use the term PPN in this manuscript for the purpose of clarity and connection to past literature.

## 3 Results

### 3.1 Distribution of PNMT‐Immunoreactive Axons Targeting Sympathetic Regions of the Spinal Cord

**FIGURE 1:**
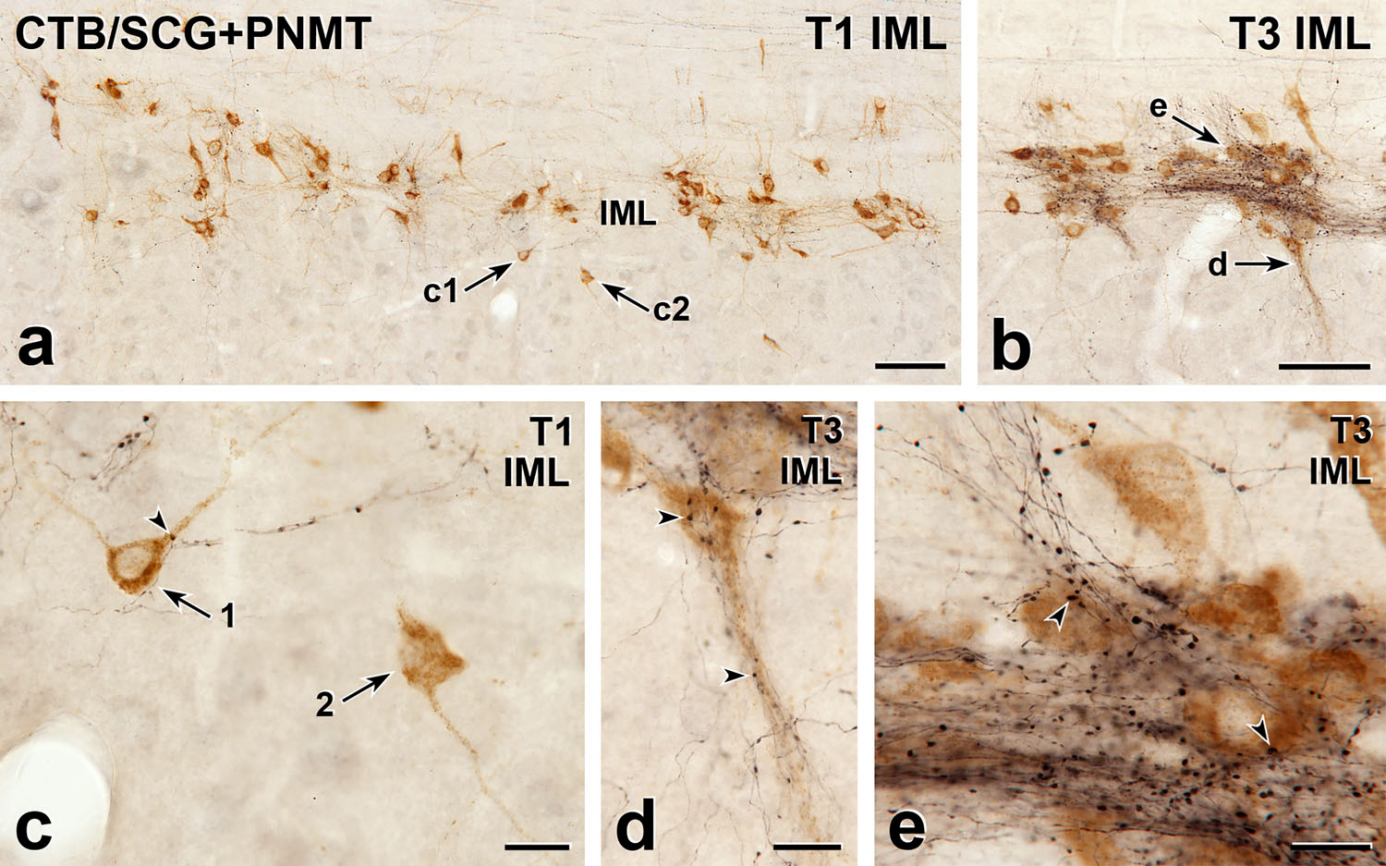
Axons immunoreactive for phenylethanolamine N‐methyltransferase (PNMT) innervate sympathetic preganglionic neurons (SPN) retrogradely labelled with cholera toxin B subunit (CTB) from the superior cervical ganglion (SCG). Horizontal sections with rostral to the left and lateral to the top. (a) In the intermediolateral cell column (IML) of thoracic segment T1, black, PNMT‐stained axons are sparsely distributed amongst brown SPN that have retrogradely transported CTB from the SCG. Scale bar, 100 µm. Arrows c1 and c2 point to SPN that are shown at higher magnification in panel c. (b) In the IML of segment T3, brown SPN containing CTB retrogradely transported from the SCG lie within a dense network of black, PNMT‐positive axons. Arrows d and e point to retrogradely labelled SPN that are shown at higher magnification in panels d and e. Scale bar, 100 µm. (c) Arrow 1 points to a brown SCG‐projecting SPN that receives a close apposition (arrowhead) from a varicosity on a black PNMT‐immunoreactive axon in the IML of segment T1. The brown, CTB‐positive SPN indicated by arrow 2 lacks any PNMT input. Scale bar, 20 µm. (d) In segment T3, a brown SCG‐projecting SPN, which has a cell body that lies at the medial edge of the IML and a major dendrite that extends medially toward the central canal, receives close appositions from black, PNMT‐positive varicosities. Some of the appositions are indicated by arrowheads. Scale bar, 20 µm. (e) In the T3 IML, many black PNMT‐immunoreactive axons surround a group of brown, CTB‐positive SPN. Arrowheads indicate examples of PNMT‐positive varicosities that closely appose cell bodies of SCG‐projecting SPN. Scale bar, 20 µm.

**FIGURE 2:**
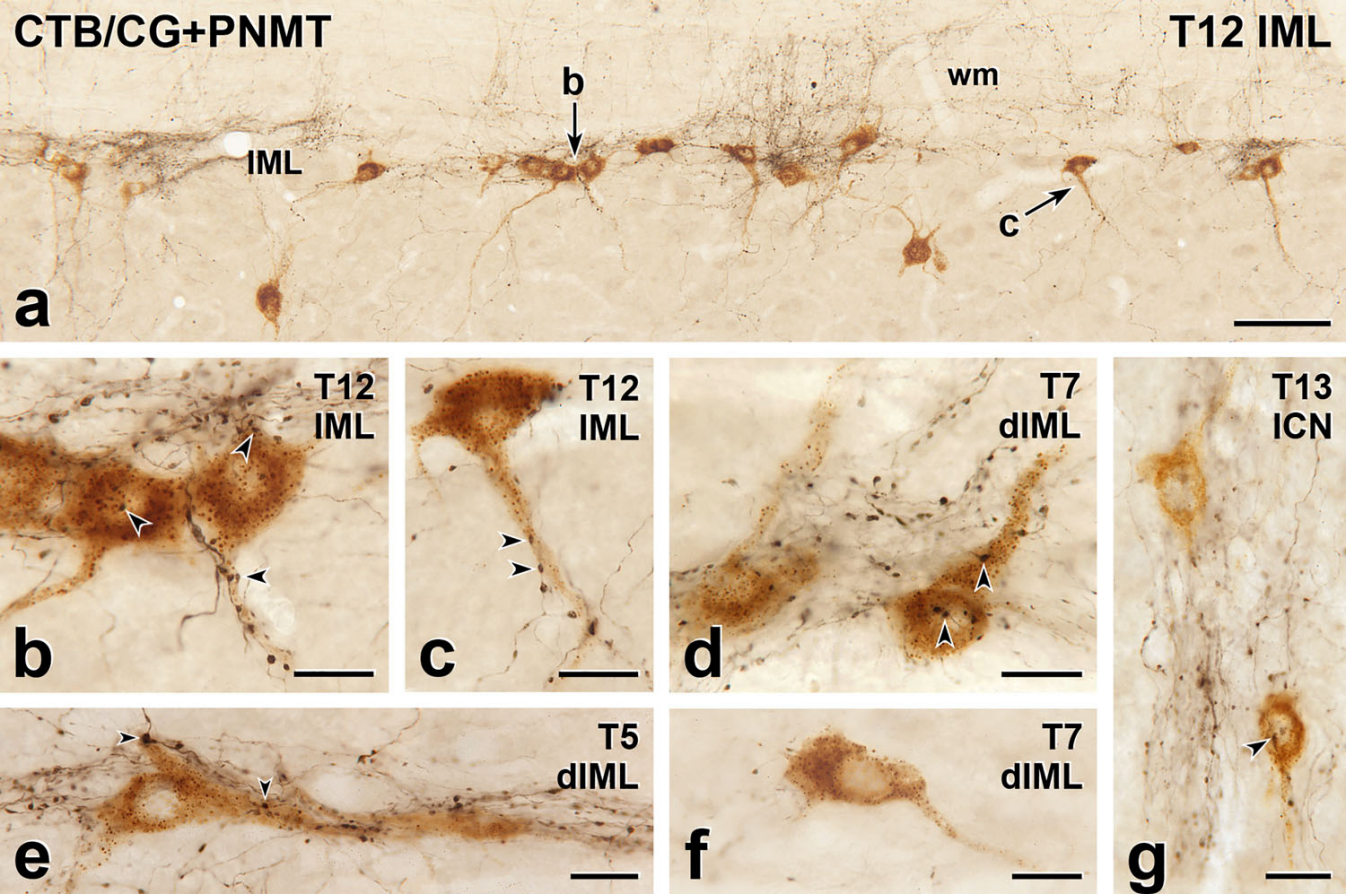
Axons immunoreactive for phenylethanolamine N‐methyltransferase (PNMT) innervate sympathetic preganglionic neurons (SPN) retrogradely labelled with cholera toxin B subunit (CTB) from the coeliac ganglion (CG). Horizontal sections with rostral to the left and lateral to the top. (a) In thoracic segment T12, black, PNMT‐labelled axons form moderately dense plexuses around nests of brown CTB‐labelled SPN in the intermediolateral cell column (IML). Individual SPN occur medial to the IML, where PNMT‐positive axons are sparse. Arrows b and c point to SPN that are shown at higher magnification in panels b and c. wm, white matter. Scale bar, 100 µm. (b) Varicosities on black, PNMT‐positive axons closely appose the cell bodies and dendrites of brown, CG‐projecting SPN in an IML nest. Arrowheads indicate examples of close appositions. Scale bar, 20 µm. (c) Black PNMT‐positive varicosities (arrowheads) closely appose a major dendrite arising from a brown CG‐projecting SPN that lies between two SPN nests. Scale bar, 20 µm. (d–f) In the dorsal IML (dIML), the packing density of SPN containing CTB retrogradely transported from the CG is lower. As a result, it is easier to identify brown CG‐projecting SPN that receive close appositions (arrowheads) from black PNMT‐immunoreactive varicosities (d and e) as well as brown CG‐projecting SPN that lack PNMT‐appositions (f). Scale bars, 20 µm. (g) In segment T13, the intercalated nucleus (ICN), which lies between the IML and the central canal, contains brown, CG‐projecting SPN that receive close appositions from black, PNMT‐labelled varicosities. The arrowhead indicates an example. Scale bar, 20 µm.

**FIGURE 3:**
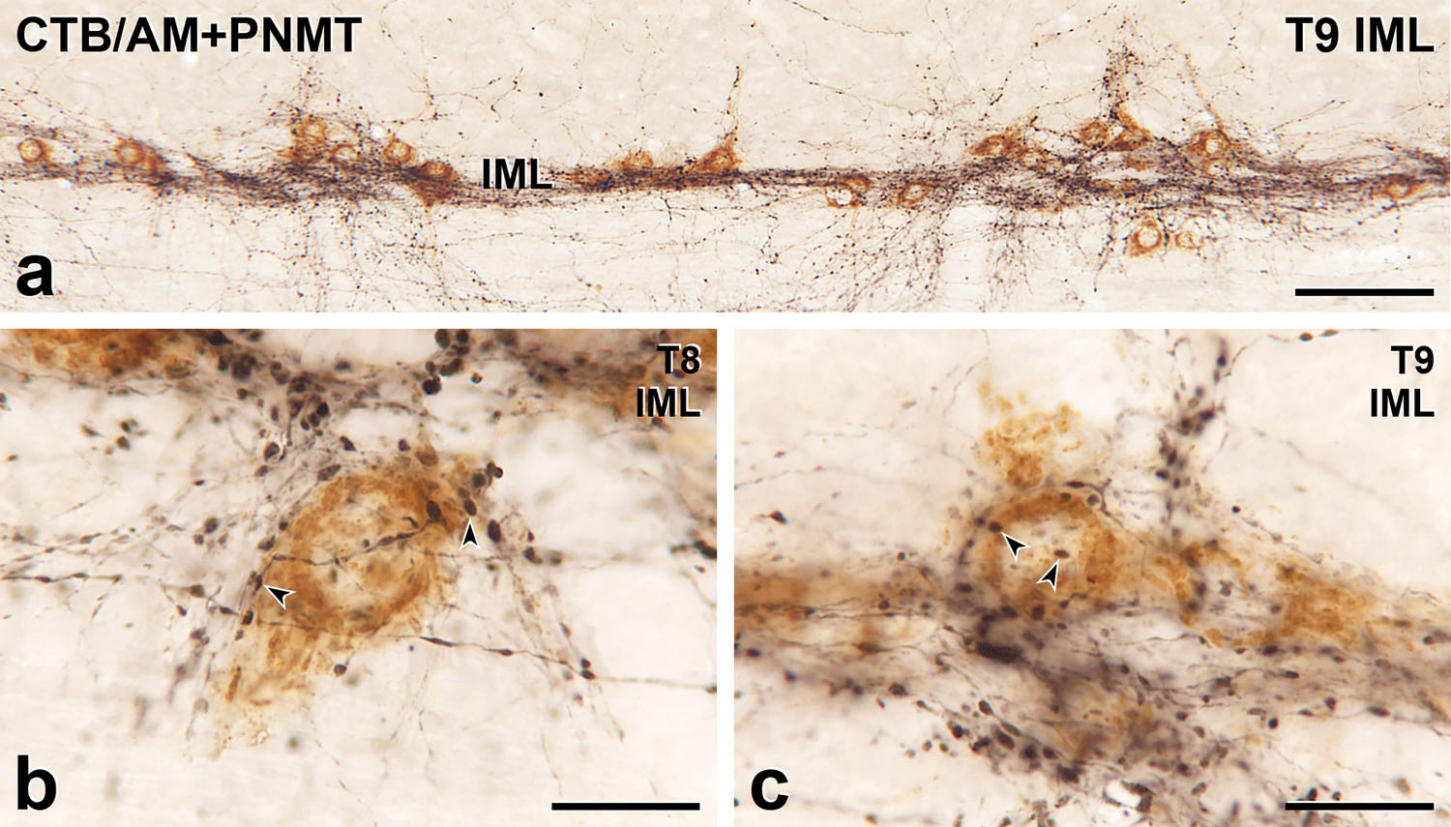
Axons immunoreactive for phenylethanolamine N‐methyltransferase (PNMT) innervate sympathetic preganglionic neurons (SPN) retrogradely labelled with cholera toxin B subunit (CTB) from the adrenal medulla (AM). Horizontal sections with rostral to the left and lateral to the top. (a) In the intermediolateral cell column (IML) of thoracic segment T9, a very dense network of black, PNMT‐labelled axons surrounds brown SPN containing CTB retrogradely transported from the AM. Scale bar, 100 µm. (b) In the IML of segment T8, the brown cell body of an AM‐projecting SPN, which is located slightly outside an SPN nest, is heavily innervated by PNMT‐immunoreactive varicosities. Arrowheads indicate examples of close appositions. Scale bars, 20 µm. (c) In the IML of segment T9, black PNMT‐labelled terminals closely appose (arrowheads) the brown cell body of an AM‐projecting SPN, which is located at the edge of an SPN nest. Scale bars, 20 µm.

**FIGURE 4:**
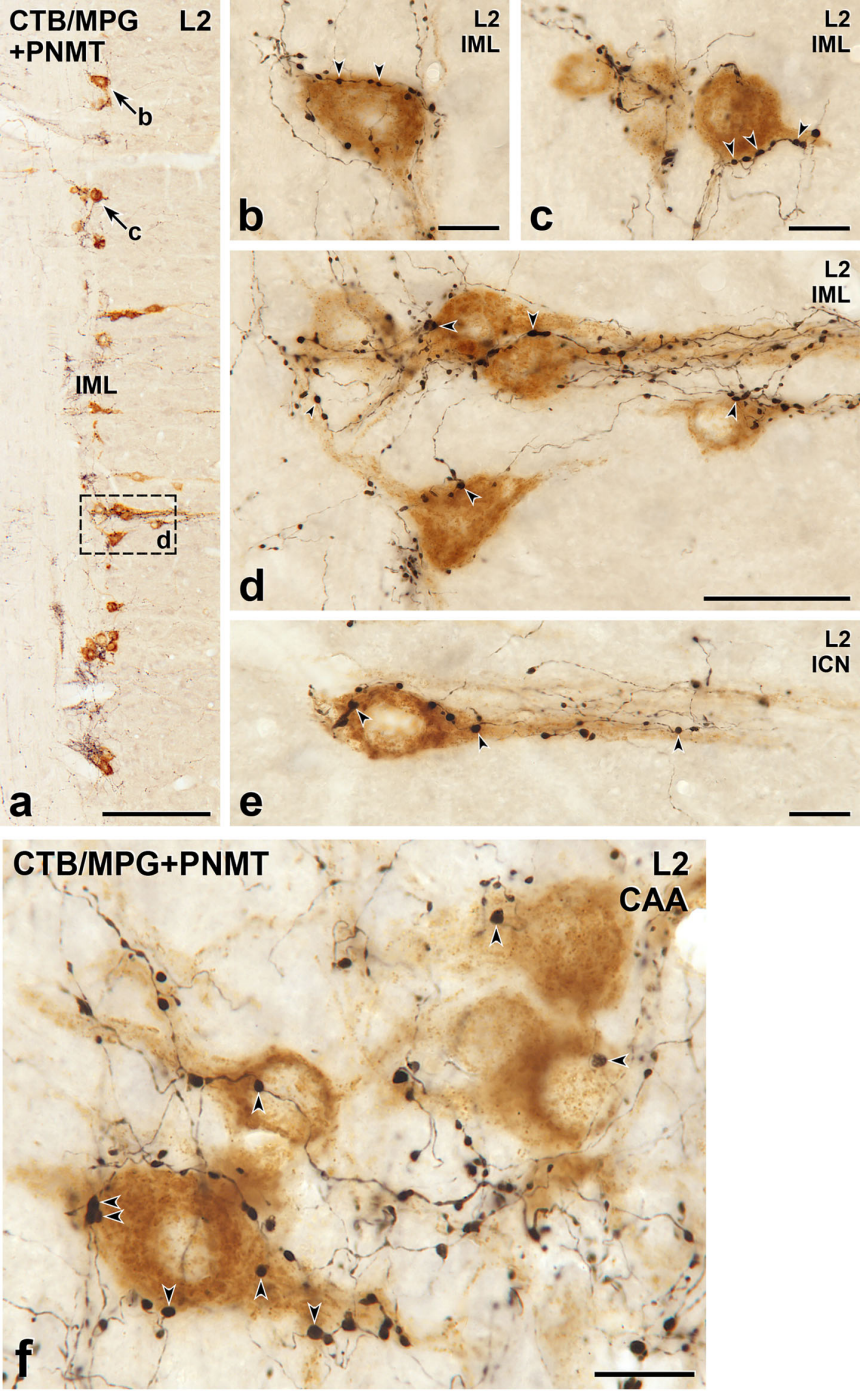
Axons immunoreactive for phenylethanolamine N‐methyltransferase (PNMT) innervate sympathetic preganglionic neurons (SPN) retrogradely labelled with cholera toxin B subunit (CTB) from the major pelvic ganglion (MPG). Horizontal sections with rostral to the top and lateral to the left. (a) In lumbar segment L2, the intermediolateral cell column (IML) contains small, closely spaced nests of SPN stained brown due to the presence of CTB retrogradely transported from the MPG. Varicose, black PNMT‐stained axons occur around the MPG‐projecting SPN and between the nests of retrogradely labelled neurons. Arrows b and c and box d indicate MPG‐projecting SPN that are shown at higher magnification in panels b, c, and d. Scale bar, 250 µm. (b–d) In the IML of L2, black PNMT‐labelled axons closely appose (arrowheads) the cell bodies and proximal dendrites of brown SPN that have retrogradely transported CTB from the MPG. Scale bars in panels b and c, 20 µm; scale bar in panel d, 50 µm. (**e**) A brown, CTB‐positive SPN supplying the MPG from the intercalated nucleus (ICN) in segment L2 receives close appositions (arrowheads) from black, PNMT‐labelled varicosities. Scale bar, 20 µm. (f) PNMT‐stained terminals innervate the cell bodies and proximal dendrites of brown, MPG‐projecting SPN in the central autonomic area (CAA) above the central canal in segment L2. Arrowheads indicate close appositions. Scale bar, 20 µm.

**FIGURE 5:**
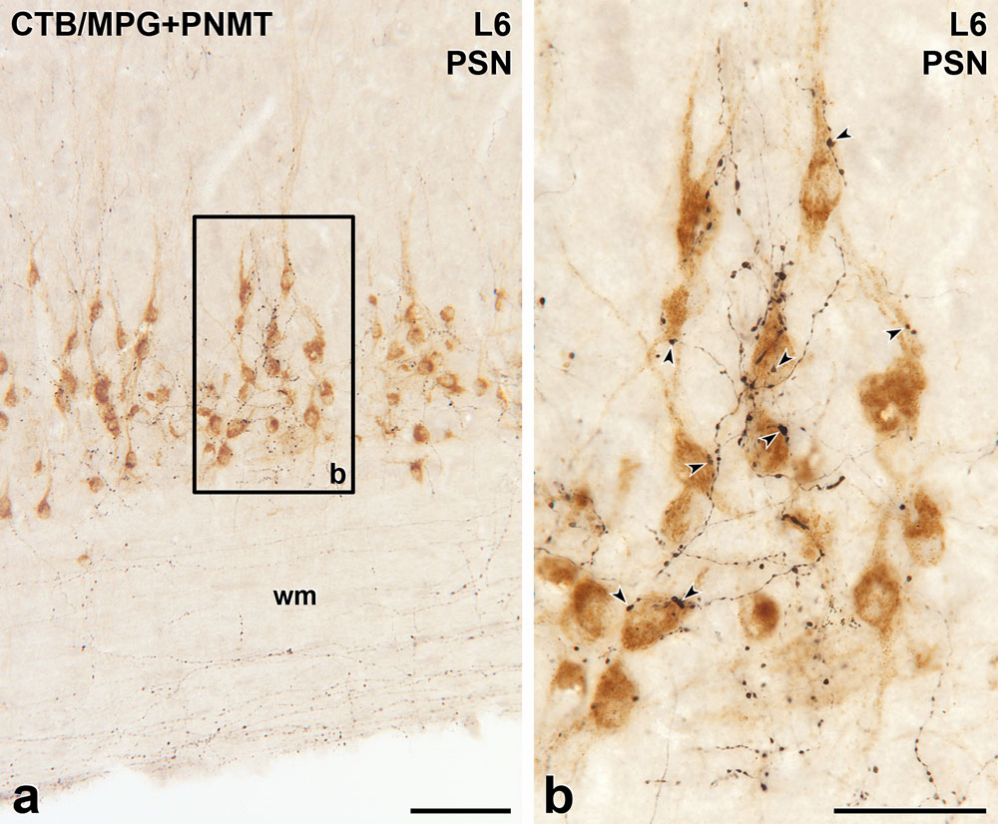
Axons immunoreactive for phenylethanolamine N‐methyltransferase (PNMT) innervate parasympathetic preganglionic neurons (PPN) retrogradely labelled with cholera toxin B subunit (CTB) from the major pelvic ganglion (MPG). Horizontal sections with rostral to the left and lateral to the bottom. (a) In lumbar segment L6, brown PPN retrogradely labelled with CTB from the MPG lie in the parasympathetic nucleus (PSN) at the lateral edge of the lower lumbar grey matter. Black PNMT‐stained axons occur around the brown PPN and also in the white matter (wm) lateral to the PSN. Box b indicates MPG‐projecting SPN that are shown at higher magnification in panel b. Scale bar, 100 µm. (b) Black, PNMT‐immunoreactive terminals closely appose (arrowheads) many of the cell bodies and proximal dendrites of brown MPG‐projecting PPN in the PSN. Scale bar, 50 µm.

Axons containing immunoreactivity for the adrenaline‐synthesizing enzyme, PNMT, occurred throughout the rostrocaudal extent of the thoracic (Figures 1, 2, 3), lumbar (Figure 4), and lumbosacral (Figure 5) segments of rat spinal cord. There were dense plexuses of PNMT‐immunoreactive axons in the intermediolateral cell column (IML) as well as in the intercalated nucleus (ICN; Figures 2 and 4) and in the central autonomic area (CAA), which lies in lamina X above the central canal. PNMT‐positive axons also travelled between these regions and were present in the white matter more caudal than spinal segment L2, where the most caudal SPN are located (Figure 5). In the grey matter as well as the white matter, the spinal PNMT‐immunoreactive axons had many varicosities.

### 3.2 PNMT‐Immunoreactive Axons Appose Retrogradely‐Labelled Sympathetic Preganglionic Neurons

The SPN were retrogradely labelled following injection of CTB into the SCG (Figure 1), CG (Figure 2), or AM (Figure 3). Injection of CTB into the MPG (Figures 4 and 5) retrogradely labelled both SPN and PPN.

The somata of SPN that supplied the SCG, CG, or MPG occurred in the IML, the ICN, and the lamina X. SPN projecting to the AM were located within the IML but not the other spinal autonomic subnuclei. The rostrocaudal distribution of SPN projecting to each of these ganglia was as described previously (Fenwick et al. 2006; Hinrichs and Llewellyn‐Smith 2009; Strack et al. 1988). Regardless of the ganglion that they innervated, the vast majority of the CTB‐immunoreactive SPN cell bodies received close appositions from PNMT‐immunoreactive axons (Figures 1, 2, 3, 4). Amongst preganglionic neurons projecting to the SCG (Figure 1c), CG (Figure 2f), or MPG (Figure 5b), we were able to detect CTB‐labelled somata that did not show evidence of PNMT input. The cell bodies and dendrites of SPN projecting to the AM are closely packed within the IML and receive a dense PNMT innervation. These structural features of AM‐projecting SPN and their adrenergic innervation meant that we were not able to identify any AM‐SPN that conclusively lacked close appositions from PNMT‐immunoreactive varicosities (Figure 3).

In the lateral horn in lower lumbar and upper sacral spinal segments, we observed clear appositions from PNMT‐immunoreactive axons onto neurons containing CTB retrogradely transported from the MPG (Figure 5). Because SPN occur only in the thoracic and upper lumbar segments of the rat spinal cord, the CTB‐labelled neurons in the lower lumbar and upper sacral segments must be PPN. Hence, this observation shows that PPN as well as SPN receive PNMT input.

### 3.3 Projections From Rostral Ventrolateral Medullary C1 Neurons

**FIGURE 6:**
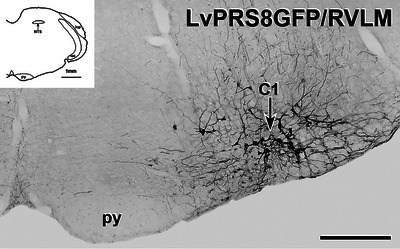
Immunolabelling for green fluorescent protein (GFP) in C1 neurons due to injection of Lv‐PRSx8‐GFP into the RVLM. Transverse section. In a section through the RVLM near the caudal pole of the facial nucleus, many C1 neurons show intense, black immunostaining for GFP after injection of Lv‐PRSx8‐GFP. Scale bar, 500 µm. *Inset*, Drawing of the section of medulla where the GFP‐immunoreactive C1 neurons shown in the micrograph were located. Scale bar, 1 mm. icp, inferior cerebellar peduncle; NTS, nucleus of the solitary tract; Py, pyramidal tract; sp5, spinal trigeminal tract.

**FIGURE 7:**
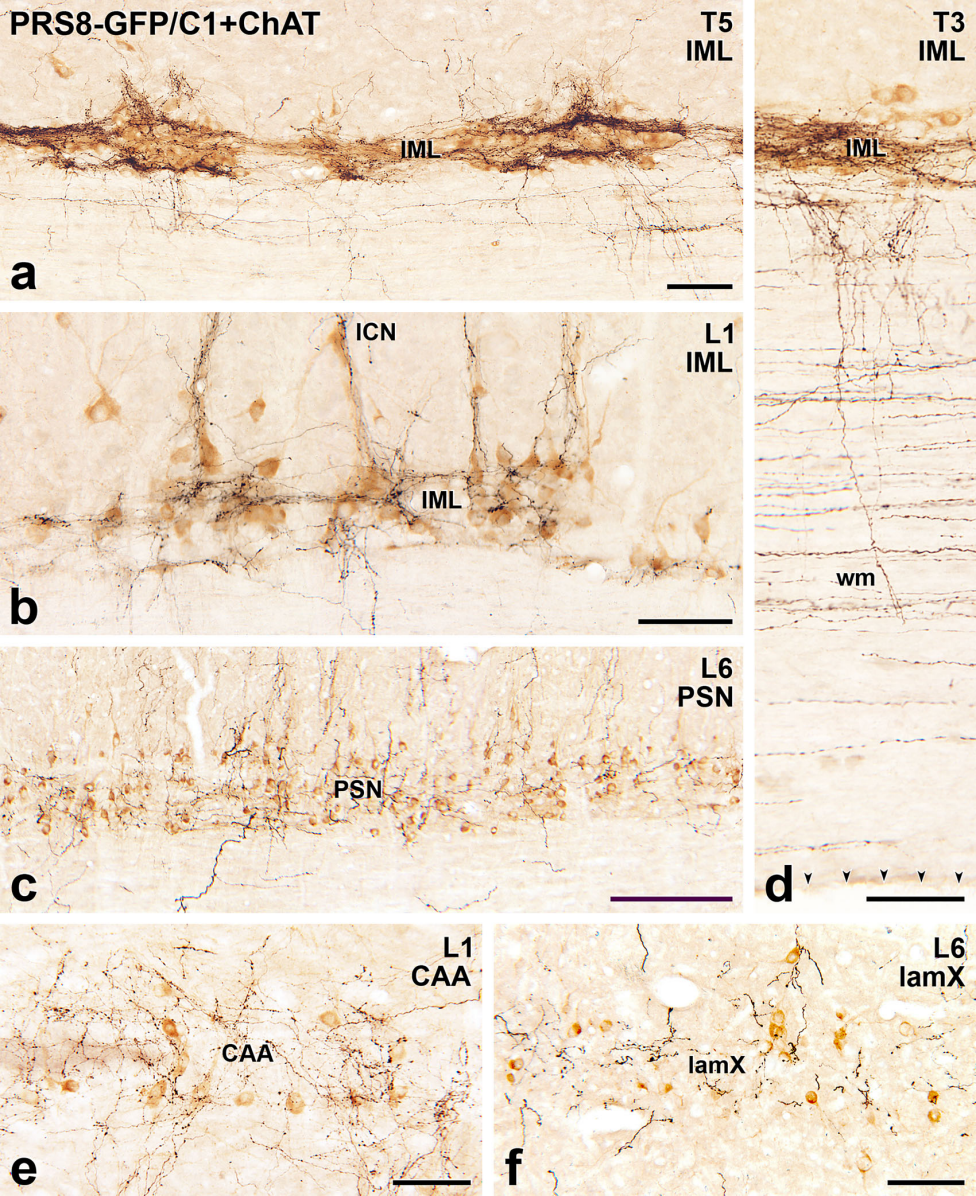
Wherever they occur in the spinal cord, preganglionic neurons immunoreactive for choline acetyltransferase (ChAT) are supplied by axons labelled with green fluorescent protein (GFP) due to Lv‐PRSx8‐GFP injections into the C1 area of the RVLM. Horizontal sections with rostral to the left and lateral to the bottom. (a) In the intermediolateral cell column (IML) of thoracic segment T5, dense plexuses of black, GFP‐stained axons are associated with nests of brown, ChAT‐positive sympathetic preganglionic neurons (SPN). GFP‐positive axons also occur in the white matter (wm). Scale bar, 100 µm. (b) In segment L1, black GFP‐containing axons surround brown ChAT‐labelled SPN in the IML and also ChAT‐labelled SPN in the intercalated nucleus (ICN, arrow). Scale bar, 100 µm. (c) In the parasympathetic nucleus (PSN) in segment L6, GFP‐immunoreactive axons travel amongst the cell bodies and dendrites of brown, ChAT‐stained parasympathetic preganglionic neurons (PPN). GFP‐stained axons also occur in the wm lateral to the PSN. Scale bar, 250 µm. (d) In segment T3 in the upper thoracic cord, many black GFP‐immunoreactive axons course through the lateral wm as well as the surrounding brown, ChAT‐positive SPN in the IML. Scale bar, 100 µm. (e) In segment L1, black, GFP‐positive axons occur around and between brown, ChAT‐stained SPN in the central autonomic area (CAA). Scale bar, 100 µm. (f) In segment L6, lamina X above the central canal contains scattered, brown, ChAT‐positive neurons that are targeted by GFP‐containing axons. Scale bar, 100 µm.

**FIGURE 8:**
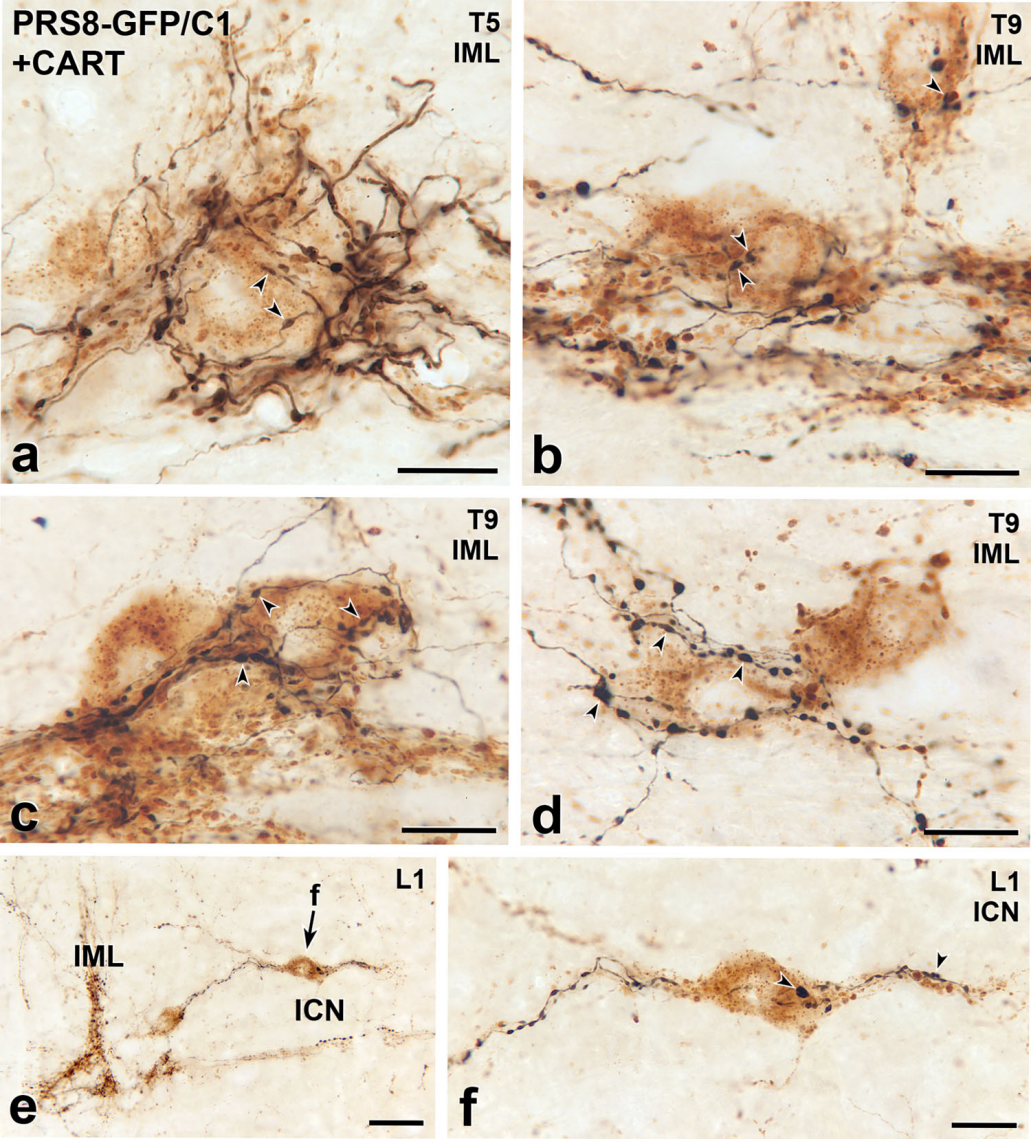
Axons immunoreactive for green fluorescent protein (GFP) due to Lv‐PRSx8‐GFP injections into the C1 area of the RVLM innervate sympathetic preganglionic neurons (SPN) immunoreactive for cocaine‐ and amphetamine‐regulated transcript (CART). Horizontal sections. (a–d) Black, GFP‐containing axons closely appose brown CART‐positive SPN in the intermediolateral cell column (IML, panels a–e) in thoracic segments T5 and T9. Scale bars, 20 µm. (**e**) Black, GFP‐labelled axons occur around brown CART‐positive SPN in the IML and also CART‐containing SPN in the intercalated nucleus (ICN). The neuron indicated by arrow f is shown at higher magnification in panel f. Scale bar, 50 µm. (f) Varicosities on a black, GFP‐stained axon closely appose (arrowheads) the cell body and proximal dendrite of a CART‐positive SPN in the ICN. Scale bar, 20 µm.

**FIGURE 9:**
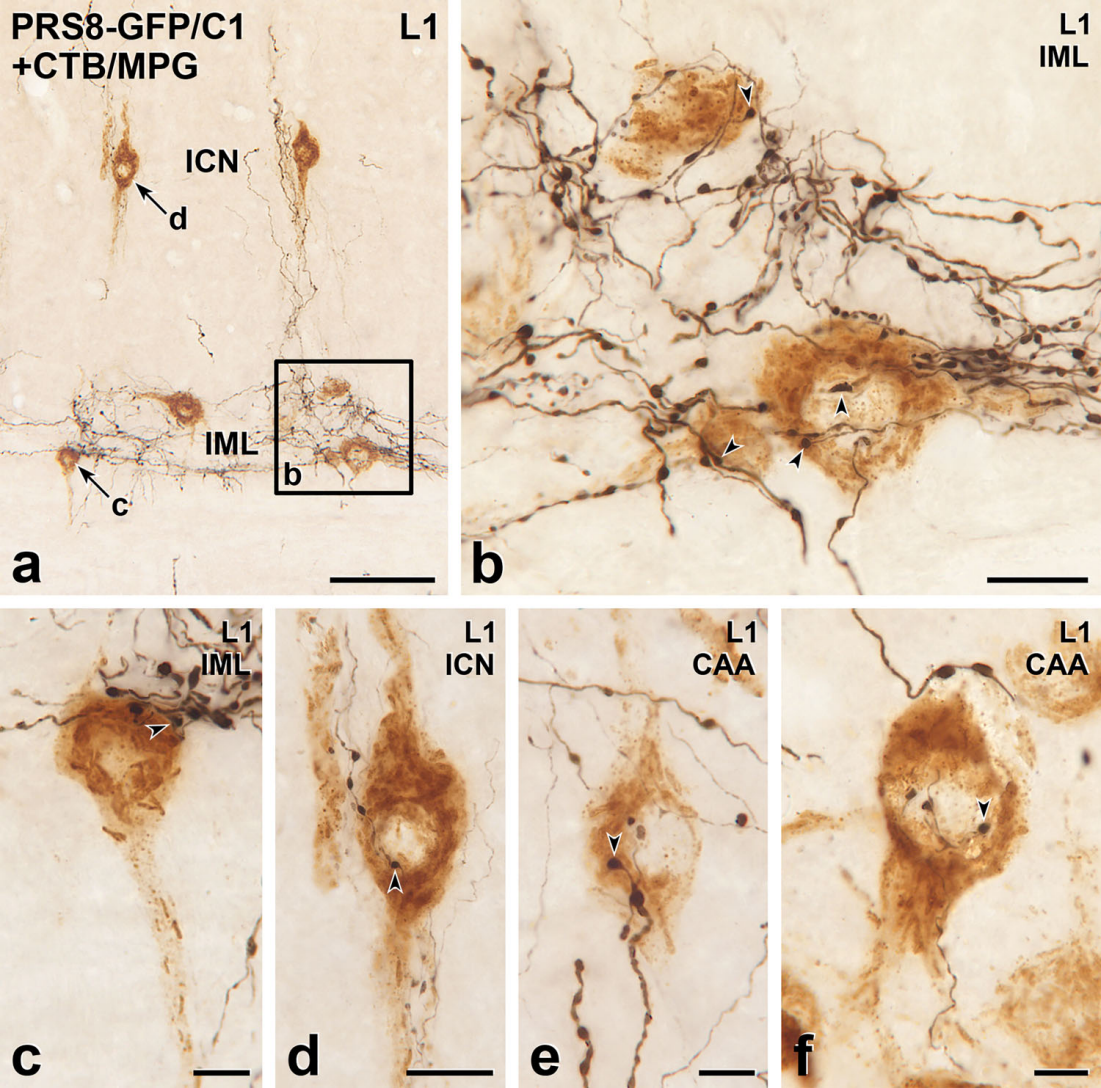
Axons labelled with green fluorescent protein (GFP) due to Lv‐PRSx8‐GFP injections into the C1 area of the RVLM innervate sympathetic preganglionic neurons (SPN) retrogradely labelled with cholera toxin B subunit (CTB) from the major pelvic ganglion (MPG). Horizontal sections with rostral to the left and lateral to the bottom. (a) In lumbar segment L1, black, GFP‐containing axons associate with brown CTB‐stained SPN in the intermediolateral cell column (IML) and in the intercalated nucleus (ICN). Boxed area b is shown at higher magnification in panel b. Arrows c and d point to retrogradely‐labelled SPN that appear at higher magnification in panels c and d. Scale bar, 100 µm. (b and c) In the IML of L1, brown SPN retrogradely labelled with CTB from the MPG receive close appositions (arrowheads) from black, GFP‐immunoreactive varicosities. Scale bar in panel b, 20 µm; scale bar in panel c, 10 µm. (d) In the ICN, a black, GFP‐immunoreactive axon closely apposes the cell body of a brown MPG‐projecting SPN. Scale bar, 20 µm. (e and f) The central autonomic area (CAA) also contains brown SPN that are retrogradely labelled with CTB from the MPG and receive close appositions (arrowheads) from varicosities on black, GFP‐stained axons. Scale bars, 10 µm.

**FIGURE 10:**
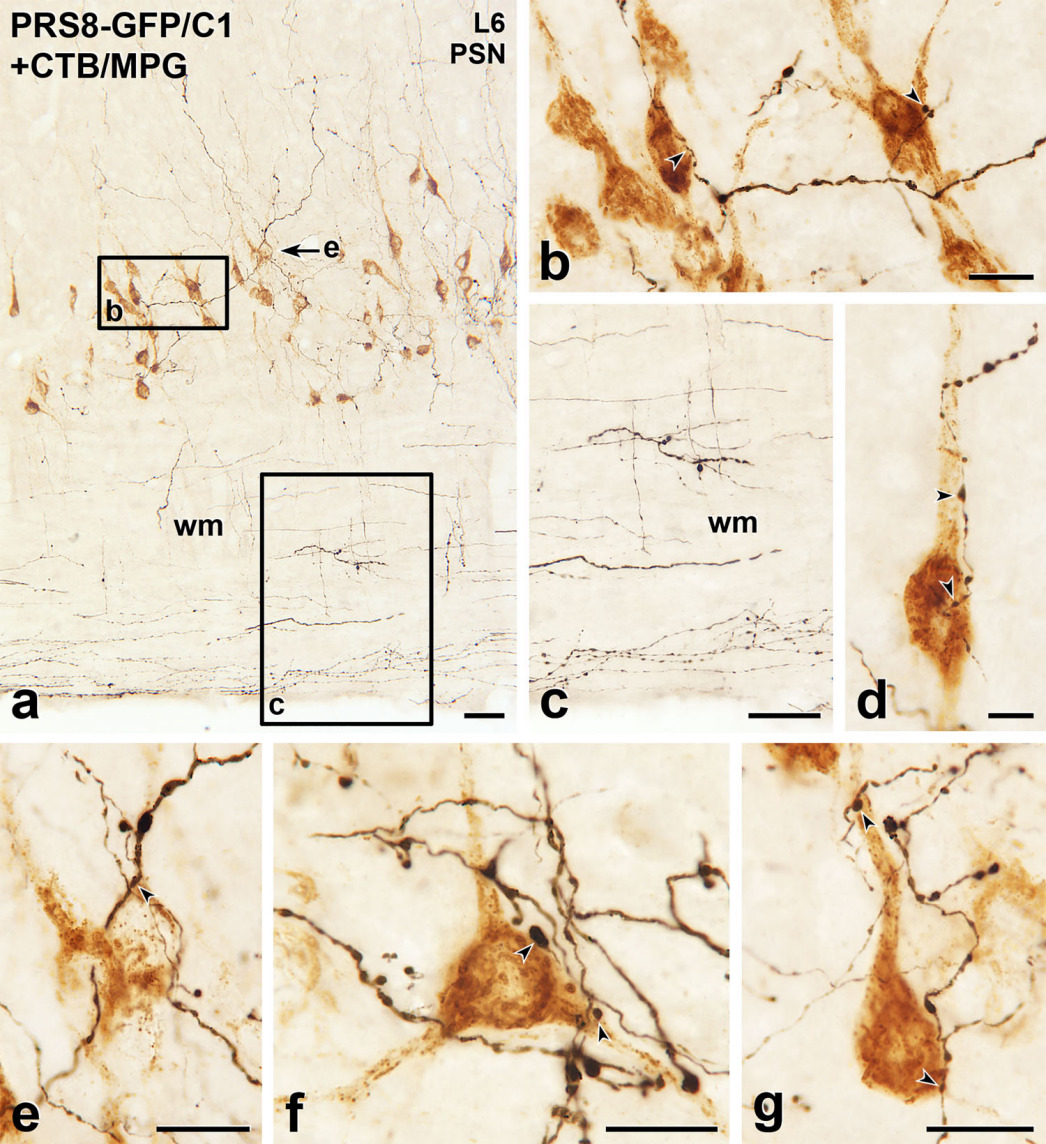
Axons labelled with green fluorescent protein (GFP) due to Lv‐PRSx8‐GFP injections into the C1 area of the RVLM innervate parasympathetic preganglionic neurons (PPN) retrogradely labelled with cholera toxin B subunit (CTB) from the major pelvic ganglion (MPG). Horizontal sections with rostral to the left and lateral to the bottom. (a) In lumbar segment L6, black, GFP‐containing axons are distributed through the parasympathetic nucleus (PSN) and come close to some brown SPN that have retrogradely transported CTB from the MPG. Black, GFP‐immunoreactive axons also occur in the grey matter medial to the PSN and in the white matter (wm) lateral to the PSN. Boxed areas b and c are shown at higher magnification in panels b and c. Arrow e indicates a retrogradely labelled PPN that appears at higher magnification in panel e. Scale bar, 100 µm. (b) Two of the brown, MPG‐projecting PPN shown in box b in panel a receive close appositions (arrowheads) from GFP‐labelled axons. Scale bar, 20 µm. (c) A higher power view of boxed area c in panel a shows varicose and non‐varicose axons that are marked by black immunostaining for GFP travelling through the white matter lateral to the PSN. Scale bar, 50 µm. (d) Varicosities on a GFP‐immunoreactive axon form close appositions (arrowheads) on the cell body and proximal dendrite of a PPN that shows brown labelling for CTB injected into the MPG. Scale bar, 10 µm. (e) A black, GFP‐positive axon closely apposes the brown, CTB‐labelled PPN indicated by arrow e in panel a. Scale bar, 20 µm. (f and g) Other examples of brown, MPG‐projecting PPN that receive close appositions (arrowheads) from black GFP‐containing varicosities. Scale bars, 20 µm.

Axons immunoreactive for PNMT in the spinal cord could arise from C1 or C3 neurons situated in the medulla oblongata, both of which have been shown to project throughout the spinal cord (Card et al. 2006; Menuet et al. 2014; Sevigny et al. 2012). To determine whether the PNMT input to SPN and PPN originated from C1 neurons, these neurons were selectively transduced using precisely targeted microinjections of a lentivirus with a specific promoter (PRSx8) driving expression of GFP (Figure 6). Axons labelled for GFP were observed throughout the spinal cord in a pattern similar to that described above for PNMT‐immunoreactive axons (Fig 7, 8, 9, 10). The C1 axons made close appositions with ChAT‐stained SPN in the IML, ICN, and CAA throughout the thoracic and upper lumbar segments, as well as with ChAT‐labelled PPN in the lateral horn of lower lumbar and sacral segments (Figure 7). Like PNMT‐positive axons, we observed GFP‐containing C1 axons in the white matter throughout the rostrocaudal extent of the spinal cord (Figure 7d).

The neuropeptide, CART, occurs in about half of SPN that express Fos in response to hypotension (Fenwick et al. 2006). We observed numerous close appositions between GFP‐labelled C1 axons and CART‐immunoreactive neurons in the IML at all thoracic and upper lumbar levels of the spinal cord (Figure 8). While not all CART‐immunoreactive neurons received C1 input, many had GFP‐positive varicosities that closely apposed their soma and/or dendrites.

The axons of C1 neurons travelled into the lumbar and upper sacral segments, where GFP‐labelled varicosities made close appositions with lateral horn neurons retrogradely labelled with CTB from the MPG (Figures 9 and 10). The varicosities of C1 axons were closely apposed to MPG‐projecting SPN in the IML, ICN, and CAA (Figure 9). In the lateral horn of segments L6 and S1, the GFP‐labelled axons of C1 origin made close appositions with PPN retrogradely labelled with CTB from the MPG (Figure 10). Even at these very distal spinal levels, we still found varicose GFP‐positive axons within the white matter (Figure 10a,c).

### 3.4 Adrenergic Inputs to Parasympathetic Preganglionic Neurons Activated by the Micturition Reflex

**FIGURE 11:**
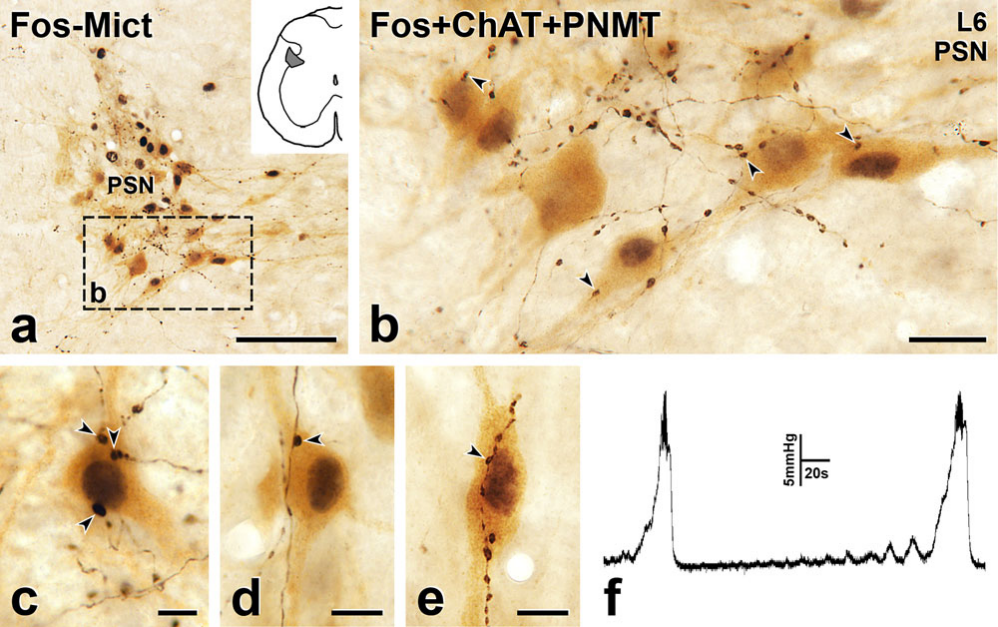
Axons immunoreactive for phenylethanolamine N‐methyltransferase (PNMT) innervate ChAT‐immunoreactive parasympathetic preganglionic neurons (PPN) that express Fos after micturition reflexes. Transverse sections. (a) In the parasympathetic nucleus (PSN) in L6 from a rat in which micturition reflexes were evoked by infusion of saline into the bladder, many brown, ChAT‐immunoreactive PPN have black, Fos‐immunoreactive nuclei. The area in box b is shown at higher magnification in panel b. Scale bar, 100 µm. (b) Black, PNMT‐labelled axons occur around and between the brown, ChAT‐immunoreactive PPN cell bodies, some of which contain black, Fos‐stained nuclei due to their activation by micturition reflexes. Some varicosities on black PNMT‐positive axons (arrowheads) appose neurons with brown, ChAT‐positive somata and black Fos‐positive nuclei. Scale bar in panel b, 20 µm. (c–e) Varicosities on black, PNMT‐immunoreactive axons (arrowheads) closely appose cell bodies of ChAT‐immunoreactive PPN that have Fos‐immunoreactive nuclei. Scale bars in panels c–e, 10 µm. (f) Trace of bladder pressure showing two consecutive micturition reflexes evoked by infusing saline into the bladder of a rat whose lumbosacral spinal cord was processed for immunolocalization of Fos and ChAT in PPN. Fos‐Mict, Fos evoked by micturition reflexes.

**FIGURE 12:**
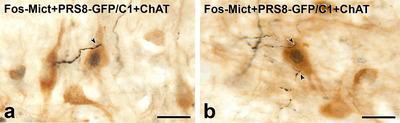
Axons labelled with green fluorescent protein (GFP) due to Lv‐PRSx8‐GFP injections into the C1 area of the RVLM innervate ChAT‐immunoreactive parasympathetic preganglionic neurons (PPN) that express Fos after micturition reflexes. Transverse sections. (a and b) Varicosities of black axons immunoreactive for GFP due to RVLM injection of Lv‐PRSx8‐GFP appose (arrowheads) brown, ChAT‐immunoreactive PPN. The PPN with GFP appositions have black, Fos‐immunoreactive nuclei, indicating that they have been activated by infusion of saline into the bladder to produce micturition reflexes. Scale bars, 50 µm.

Lumbosacral PPN play a key role in the micturition reflex (de Groat et al. 2015). In response to increased bladder volume, this sensory reflex results in activation of the PPN, resulting in constriction of the bladder and urethral smooth muscle to promote voiding. We induced the micturition reflex in anesthetized rats and used Fos immunoreactivity in lumbosacral PPN to identify those involved in controlling the micturition reflex (Figure 11). Infusion of saline into the bladder resulted in a gradual increase in bladder pressure that led to a sharp increase in pressure associated with activation of the micturition reflex and voiding (Figure 11f). Micturition reflexes evoked over a 2‐h period induced Fos expression in ChAT‐immunoreactive PPN in the lumbar parasympathetic nucleus (Figure 11a). These Fos‐immunoreactive neurons received close appositions from PNMT‐stained varicosities (Figure 11b–e). In separate rats with bladder infusion of saline and lentiviral PRSx8‐GFP injections into the C1 region, we also observed close appositions between GFP‐stained C1 axons and Fos‐positive PPN that had been activated by the micturition reflex (Figure 12).

### 3.5 Adrenergic Inputs to Somatic Motor Neurons in the Ventral Horn

**FIGURE 13:**
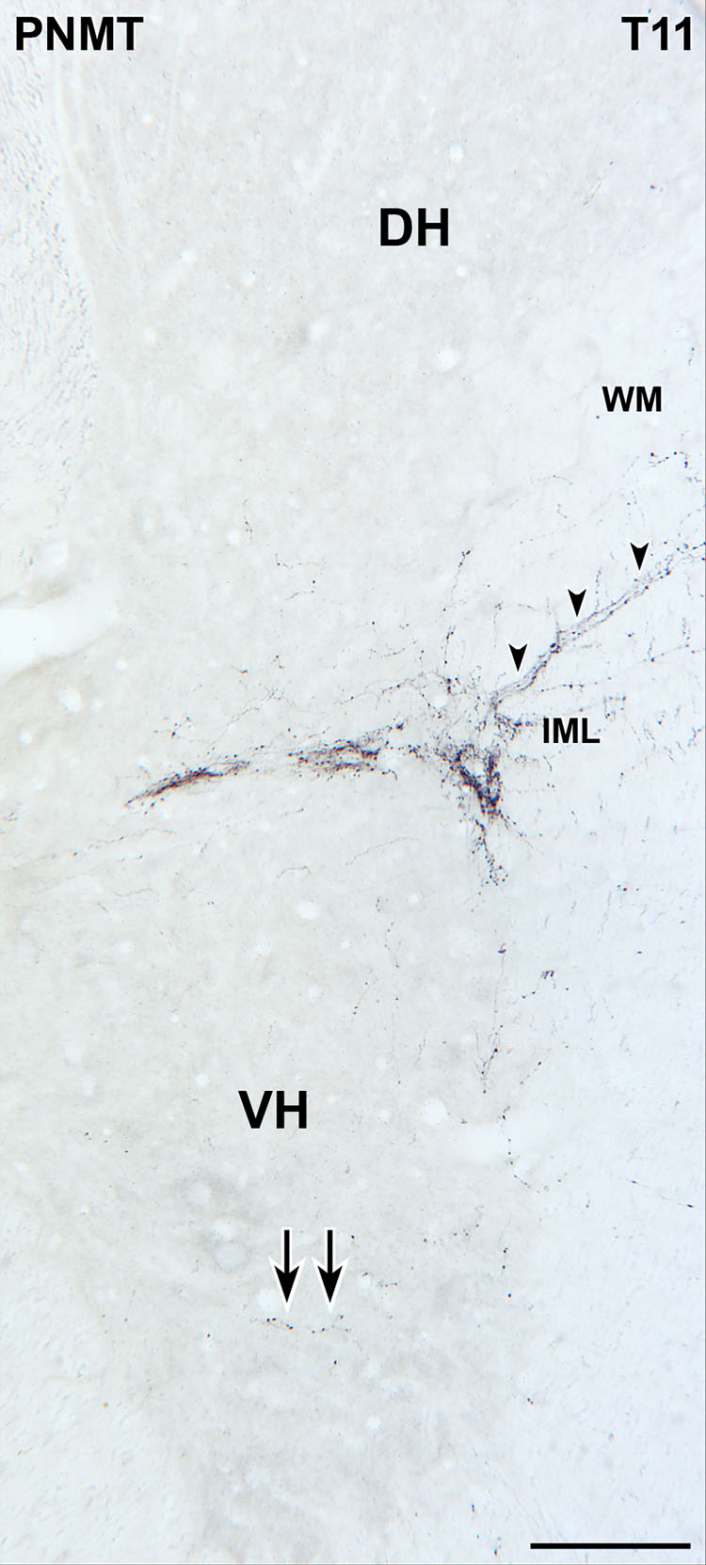
The ventral horn (VH) contains axons immunoreactive for phenylethanolamine N‐methyltransferase (PNMT). Transverse section. A few black, varicose, PNMT‐immunoreactive axons (arrows) occur in the ventral horn of the thoracic spinal cord segment T11. The intermediolateral cell column (IML) receives a dense PNMT‐stained innervation. Black PNMT‐containing axons also travel in the lateral white matter (WM) toward the IML, probably following the dendrites of SPN that project into this region. Scale bar, 50 µm. DH, dorsal horn.

**FIGURE 14:**
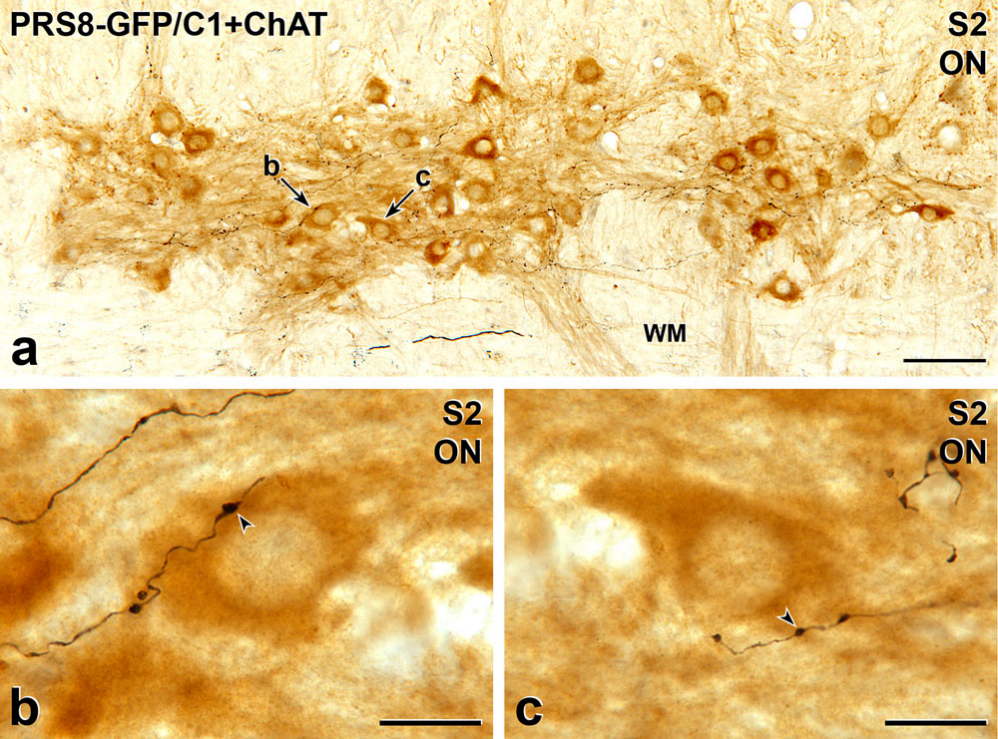
Axons labelled with green fluorescent protein (GFP) due to Lv‐PRSx8‐GFP injections into the C1 area of the RVLM innervate ChAT‐immunoreactive motor neurons in Onuf's nucleus (ON). Horizontal sections. (a) ON in sacral segment S2 contains many motor neurons stained brown due to the presence of ChAT‐immunoreactivity. Occasionally, black, varicose GFP‐immunoreactive axons wander amongst the brown‐stained motor neurons. Black, GFP‐labelled axons also occur in the white matter (WM) lateral to ON. The motor neurons indicated by arrows b and c are shown at higher magnification in panels b and c, respectively. Scale bar, 100 µm. (b) A varicosity on a black, GFP‐labelled axon forms a close apposition (arrowhead) on the brown cell body of a ChAT‐stained motor neuron in ON. Scale bar in panel b, 20 µm. (c) A varicosity on a black, GFP‐positive axon forms a close apposition (arrowhead) on a proximal dendrite of a brown, ChAT‐immunoreactive motor neuron in ON. Scale bar in panel c, 20 µm.

Previous studies on adrenergic projections to the spinal cord have focused on inputs to autonomic neurons in the lateral horn, the ICN, and the lamina X in thoracic and upper lumbar spinal segments. In this examination of the spinal distribution of PNMT‐immunoreactive axons and axons arising from C1 neurons, we observed occasional varicose, PNMT‐stained axons wandering amongst ventral horn motor neurons (Figure 13). These rare PNMT‐stained axons were present in the ventral horn in transverse sections from cervical segment 8 to sacral segment 4. Because of our interest in spinal neurons involved in micturition, we assessed the PNMT innervation of somatic motor neurons that innervate the external urethral sphincter to control voiding. These motor neurons lie in Onuf's nucleus in the ventral horn of sacral segments S2–S4. On the cell bodies and/or dendrites of occasional ChAT‐stained motor neurons in Onuf's nucleus, we observed close appositions from GFP‐immunoreactive C1 varicosities (Figure 14).

## 4 Discussion

This anatomical study examined the distribution of axons immunoreactive for the adrenaline‐synthesizing enzyme, PNMT, throughout the rostrocaudal extent of the spinal cord of the rat. We observed PNMT in axons at all levels of the spinal cord from the lower cervical through thoracic and lumbar to sacral segments. These axons make close appositions with SPN projecting to the SCG, CG, or MPG or to the AM, and with PPN projecting to the MPG. Using a viral transgenic approach, we demonstrated that C1 neurons of the RVLM make a major contribution to this adrenergic input, with GFP‐labelled axons from C1 neurons apposing ChAT‐immunoreactive preganglionic neurons at all levels of the spinal cord examined. Furthermore, we demonstrated that PNMT‐containing axons and GFP‐containing C1 axons make close appositions with PPN activated by the micturition reflex, as well as somatic motor neurons that are involved in bladder control and lie in Onuf's nucleus in the ventral horn of the sacral spinal cord. It is likely that at least half of these close appositions represent synapses based on our previous observations (Pilowsky et al. 1992). Surprisingly, we also observed a sparse adrenergic input to the ventral horn at most levels of the spinal cord. The neurons targeted by this input were not identified, so they could be either somatic motor neurons or spinal interneurons.

The main observation from this light microscopic study is that most SPN and many PPN receive an adrenergic input. This finding clearly points to RVLM C1, and potentially C3, neurons modulating most sympathetic and spinal parasympathetic functions. Previous studies have demonstrated extensive projections of both C1 (Card et al. 2006; Sevigny et al. 2008) and C3 (Sevigny et al. 2012) neurons throughout the rostral–caudal extent of the spinal cord. Indeed, co‐labelling of C1 and C3 neurons indicates that many SPN receive inputs from both cell groups (Menuet et al. 2014). In this study, we demonstrate that SPN retrogradely labelled from different sympathetic ganglia receive PNMT inputs from C1 neurons as do PPN retrogradely labelled from the MPG. Most SPNs in the IML, ICN, and lamina X in the thoracic and upper lumbar spinal cord receive adrenergic innervation as do a significant proportion of PPN in the parasympathetic nucleus in the lower lumbar/upper sacral cord. Our light microscopic observations suggest that occasional preganglionic neurons do not receive a PNMT or C1 input. Neurons lacking adrenergic input included some SPN that projected to the SCG, CG, or MPG, and some PPN projecting to the MPG. Nevertheless, for these neurons, we cannot rule out the possibility of adrenergic input to the distal dendrites of neurons that show no somatic input.

Many SPN at most levels of the thoracolumbar spinal cord express CART, and this neurochemical population of neurons innervates sympathetic ganglia and the adrenal gland (Dun et al. 2000; Fenwick et al. 2006). The expression of CART is considered to define SPN with cardiovascular function (Gonsalvez et al. 2010), which are not activated by stimuli involved in glucose homeostasis (Parker et al. 2013). At all levels of the thoracolumbar spinal cord examined, CART‐immunoreactive SPN receive appositions from varicose axons arising from C1 neurons. Only rarely did we observe CART neurons that did not receive C1 input. This observation is congruent with the well‐described influence of C1 neurons on sympathetic vasomotor activity. The RVLM and its C1 adrenergic neurons are considered key contributors to the regulation of sympathetic vasomotor tone. C1 neurons receive autonomic sensory inputs and contribute to the baroreceptor and chemoreceptor regulation of sympathetic vasomotor activity (Dampney 1994; Guyenet et al. 2013). They also receive input from the central respiratory generator (Moraes et al. 2013; Moraes et al. 2014), and C1 neurons are considered a key component of the exaggerated respiratory modulation of sympathetic activity that contributes to the development of neurogenic hypertension (Menuet et al. 2020; Menuet et al. 2017).

The extensive distribution of C1 axons throughout the neuraxis (Card et al. 2006; Sevigny et al. 2017) suggests more diverse functions for this cell group. The C1 neurons are activated by metabolic stimuli (Menuet et al. 2014; Ritter et al. 1998; Senthilkumaran et al. 2018) and contribute to the regulation of glucose in response to hypoglycemia (Verberne and Sartor 2010). Direct excitatory inputs have been demonstrated from C1 neurons to noradrenergic neurons in the locus coeruleus (DePuy et al. 2013), orexinergic neurons of the lateral hypothalamus (Bochorishvili et al. 2014), and PPN in the dorsal motor nucleus of the vagus (DePuy et al. 2013). The data reported here support this evidence of more widespread neural regulation by C1 neurons.

In this study, we describe PNMT/C1 input to PPN that is activated by the micturition reflex as well as to somatic motor neurons located in Onuf's nucleus, which are involved in voiding. Blood flow to the detrusor muscle increases during micturition (Macnab et al. 2012). This observation means that bladder vasomotor preganglionic neurons would be inhibited by micturition and would not express Fos after evocation of this reflex. We therefore conclude that C1 neurons projecting to PPN activated by the micturition reflex are most likely associated with functions other than regulation of tissue blood flow. The neural circuitry regulating micturition has been reviewed in detail (de Groat et al. 2015; Fowler et al. 2008), incorporating data derived from many studies that have used transsynaptic tracing with pseudorabies virus to identify brain pathways projecting polysynaptically to the bladder and urethra (Nadelhaft and Vera 1995, 1996, 2001; Nadelhaft et al. 1992; Vizzard et al. 1995). With the exception of some uncharacterized neurons projecting to the urethral sphincter that are located in the region of the RVLM (Nadelhaft and Vera 1996), none of these studies indicate a projection from C1 neurons, despite evidence presented here of close appositions. Previous studies have described a descending catecholaminergic input to the circuits that regulate the urinary tract. This input is considered to arise from the locus coeruleus and A5 nucleus and is proposed to play a nonspecific, level‐setting function (Holstege 2005). Further examination of the input from the C1 neurons identified here may be important for understanding regulation of bladder control in health and disease, particularly as C1 neurons are known to be affected in conditions, such as Parkinson's Disease (Gai et al. 1993), where bladder dysfunction is common (Sakakibara et al. 2012).

While PNMT inputs clearly preferentially target autonomic neurons, our observation of adrenergic innervation of Onuf's nucleus prompted a more extensive and careful examination of PNMT‐immunoreactive axon terminals in other somatic motor nuclei. We found evidence of PNMT and C1 inputs to neurons in the ventral horn at many levels of the spinal cord. These adrenergic axons were very sparse compared to the extremely high‐density surrounding SPN. Whether the innervated neurons in the thoracolumbar ventral horn are somatic motor neurons or spinal interneurons, we did not determine. The functional relevance of an adrenergic input to ventral horn neurons is not known. Microinjections of noradrenaline into the ventral horn potentiate evoked c‐fiber reflex activation of motor outputs in anesthetized cats (Bell and Matsumiya 1981). In the neonatal mouse spinal cord, noradrenaline modulates induced locomotor, or ventral root, activity, with the direction of modulation dependent upon the receptor subtype activated—clonidine suppressing and phenylephrine activating the response (Gordon and Whelan 2006; Zimmerman et al. 2012). In anesthetized rats, noradrenaline has only a small effect on somatic motor neuron activity but facilitates reflex glutamatergic inputs (Schwarz et al. 2008). We are not aware of studies specifically examining an adrenergic influence on the function of somatic motor neurons or spinal interneurons. While we show evidence for adrenergic input here, it should be remembered that C1 neurons are glutamatergic neurons (DePuy et al. 2013; Guyenet et al. 2013), so our study also reveals an excitatory bulbospinal input to SPN and PPN. We speculate that the adrenergic input to the ventral horn described in this study is functionally relevant, and future studies might investigate whether this is one mechanism by which autonomic and motor functions are coordinated.

This study provides a comprehensive map of the distribution of adrenergic axons, including those from medullary C1 neurons, from the lower cervical to lower sacral spinal cord of the rat. Our evidence shows that adrenergic inputs are anatomically placed to influence most sympathetic and parasympathetic output from the spinal cord. While this influence includes well‐established roles in cardiovascular function, the innervation described here suggests that C1 neurons may modulate a broader range of spinal function than previously presumed, including micturition and potentially some somatic motor functions.

## Conflicts of Interest

The authors declare no conflicts of interest.

## Data Availability

The original images displayed in this manuscript are available from the corresponding author upon reasonable request.
